# Microparticle Bombardment as a Method for Transgenesis in *Auanema* and *Tokorhabditis*

**DOI:** 10.17912/micropub.biology.001585

**Published:** 2025-05-08

**Authors:** Tatsuya Yamashita, Andre Pires-daSilva, Shun Oomura, Taichi Kusano, Nami Haruta, Mayu Hasumi, Taisei Kikuchi, Sally Adams, Asako Sugimoto, Ryoji Shinya

**Affiliations:** 1 School of Agriculture, Meiji University, Kanagawa, Japan; 2 School of Life Sciences, University of Warwick, Gibbet Hill, England, United Kingdom; 3 Graduate School of Life Sciences, Tohoku University, Miyagi, Japan; 4 Graduate School of Frontier Sciences, The University of Tokyo, Chiba, Japan

## Abstract

Functional gene analysis tools in *Caenorhabditis elegans* are often ineffective in other nematodes due to differences in gonadal morphology and transgene silencing. Here, we established a method to generate stable transgenic lines in the nematodes *Auanema freiburgense* and *Tokorhabditis tufae* using microparticle bombardment coupled with hygromycin B selection. Despite using non-codon-optimized GFP, transgenic strains expressing fluorescent markers were obtained in both species. Additionally, an *Auanema* codon-optimized RFP construct showed robust expression in all tissues. This method will be valuable for future studies into the unusual sex determination, viviparity, and stress resistance in *Auanema* and *Tokorhabditis* .

**Figure 1. Ballistic bombardment-generated transgenic strains:**
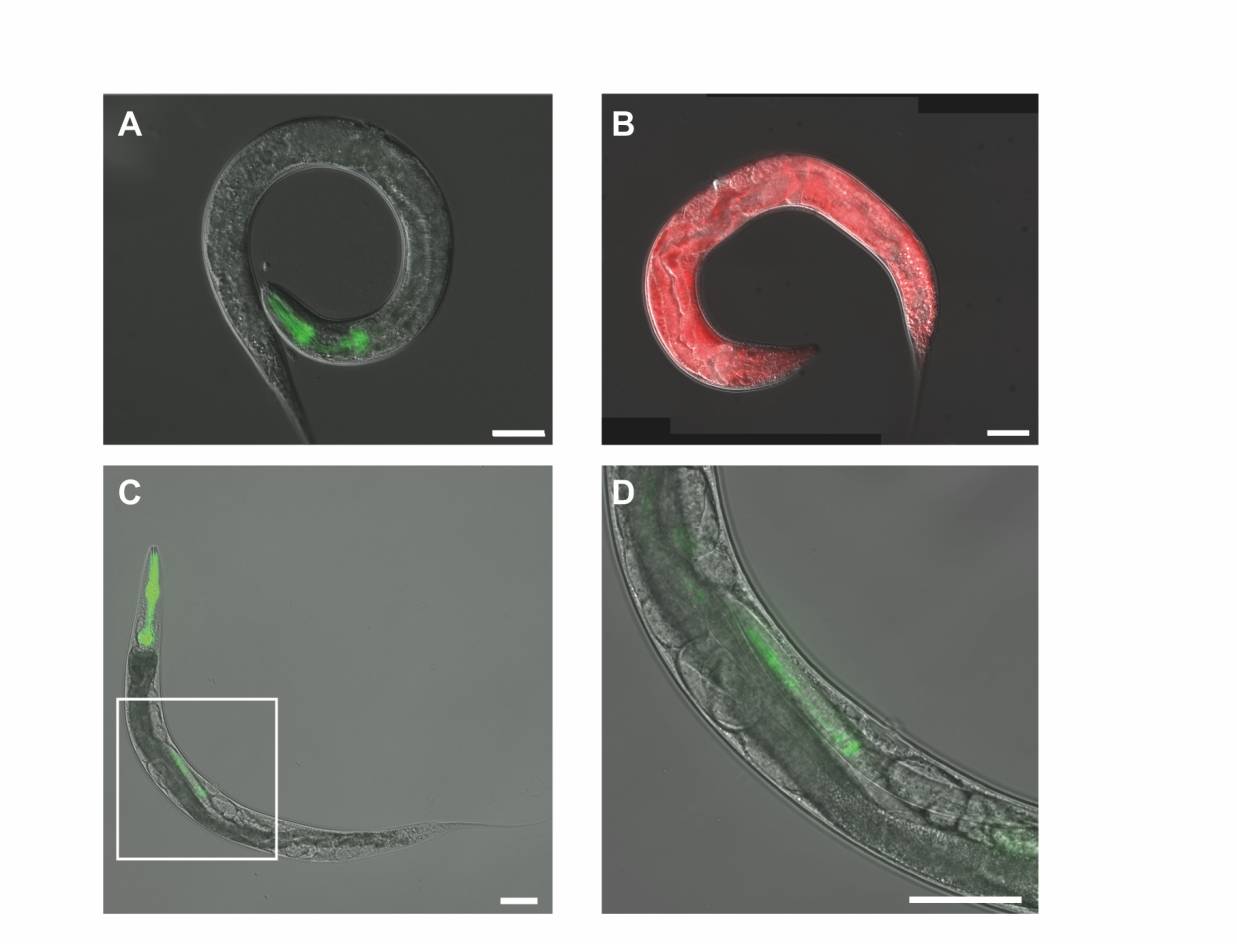
( **A** ) *A. freiburgense* hermaphrodite with pharyngeal *myo-2 p* ::GFP expression and ( **B** ) ubiquitous *eft-3p* ::RFP expression. ( **C** ) A low-magnification image of *T. tufae* with a square inset highlighting a region shown at higher magnification in ( **D** ). Pharyngeal *myo-2 p::GFP* expression is visible in the adult worm ( **C** ) and its offspring within the uterus ( **D** ). Scale bar: 50 µm.

## Description

While tools for functional gene analysis are well established in the model nematode *Caenorhabditis elegans* , many are not broadly applicable across Nematoda. For instance, stable transgenic lines in *C. elegans* are readily generated via intragonadal microinjection, typically forming extrachromosomal arrays that are transmitted for multiple generations. However, in non- *Caenorhabditis* nematodes, arrays are often silenced in the F2 and subsequent generations (Higazi et al., 2002; Junio et al., 2008; Schlager et al., 2009). These and other technological difficulties limit the exploration of the diverse biology of nematodes (Félix, 2008).

The free-living nematodes *Auanema* and *Tokorhabditis* , sister taxa within Rhabditidae (Sudhaus et al., 2023), have attracted interest due to their unusual mating system with males, females, and hermaphrodites (Félix, 2004; Chaudhuri et al., 2011), their ability to survive in extreme environments (Shih et al., 2019; Kanzaki et al., 2021), and obligate viviparity (Yamashita et al., 2023) (for a recent review, see (Adams et al, 2025)). Although CRISPR/Cas9 and RNAi methods have been established in *Auanema* (Adams et al., 2019), achieving stable transgenesis remains challenging. The relatively small, non-syncytial gonads of *Auanema* and *Tokorhabditis* make gonad microinjections difficult, and the F1 generation does not transmit transgenes to the next generation (Adams et al., 2019).

Microparticle bombardment is an alternative method for generating transgenes, often resulting in chromosomal integration and thus more stable transformations (Jackstadt et al., 1999; Higazi et al., 2002; Semple et al., 2012; Radman et al., 2013; Semple and Lehner, 2014; Namai and Sugimoto, 2018; Oomura et al., 2022). For instance, in *C. inopinata* , which is technically challenging to microinject due to its small and fragile gonads, microparticle bombardment enabled the generation of stable transformants (Oomura et al., 2022).

Here, we established the microparticle bombardment combined with the hygromycin B selection method for the representative species *A. freiburgense* and *T. tufae* . The protocol for *Auanema* and *Tokorhabditis* was based on the protocols for *C. elegans* (Schweinsberg and Grant, 2013) and *P. pacificus* (Namai and Sugimoto, 2018) with some modifications (Methods). To obtain the 100,000 *A. freiburgense* adult individuals required for bombarding one DNA construct, we harvested worms from unsynchronized cultures of 20 large (90 mm) enriched NGM (2.5% agar) plates. After bombardment, worms were incubated at 25 °C and submitted to selection by adding hygromycin 24 hours after bombardment. To collect *T. tufae* adult worms for the bombardment, we first synchronized the culture by collecting dauer larvae using a 1 % SDS solution. After washing out the solution, dauers were grown at 20 °C for 44-48 hours until they became adults. Hygromycin B was added to the plate of *T. tufae* 4 days after bombardment.

Using a *myo-2* promoter-driven GFP construct, we established two independent transgenic *A. freiburgense* lines and one *T. tufae* line. The efficiency of obtaining stable transgenic lines was approximately one line per 100,000 bombarded worms for *A. freiburgense* and one line per 45,000 worms for *T. tufae* . Notably, despite the GFP construct not being codon-optimized for either species, we observed successful transgenic expression in both. As expected, GFP expression was localized to the pharynx ( Fig. 1A, C). In *T. tufae* , a viviparous species, fluorescence was also detected in developing larvae within the maternal uterus ( Fig. 1D ). An *Auanema* codon-optimized RFP driven by the ubiquitous *eft-3* promoter was equally effective, showing expression in all adult tissues ( Fig. 1B ).

This study successfully established transformation techniques for *T. tufae* and *A. freiburgense* . To maximize transformation efficiency in each nematode species, future studies should compare codon-optimized plasmids. While these nematodes belong to the same family as *C. elegans* , they have distinct ecological and biological traits. The development of transgenics in these species will be crucial for future studies in studying the evolution of sex determination, viviparity, and stress resistance.

## Methods

***A. freiburgense* and *T. tufae* culture**

The wild type *A. freiburgense* (SB372) and *T. tufae* (PS8402) were cultured at 25 °C and 20 °C, respectively, on nematode growth medium (50 mM NaCl, 0.25% bacto peptone (Gibco), 1 mM CaCl _2_ , 1 mM MgSO _4_ , 25 mM pH 6.0 KH _2_ PO _4_ , 5 μL/mL cholesterol, 2.5% agar (STAR), and seeded with *Escherichia coli* OP50 as a food source. For *A. freiburgense* cultivation, 20 unit/mL Nystatin was added.

**Construction of vector plasmids**

The *Ppa* -TurboRFP and hygromycin B resistance gene (IR98) (Radman, 2013) coding sequences, used in plasmids pAS66 and pAS71, respectively, were codon-optimized for *Auanema* using a method previously described (Han et al., 2020). To determine the optimal codons, *A. freiburgense* RNA-seq data (PRJEB60474) was analyzed using cusp from the EMBOSS suite (Rice et al., 2000) to calculate codon usage bias for genes with different expression levels. The coding sequences of *Ppa* -TurboRFP and IR98 were subsequently modified to use the preferred codons identified in the most highly expressed genes. The codon-optimized *Ppa* -TurboRFP sequence was further engineered by the addition of three introns from the highly expressed *Afr-actin-1* homolog (Afr-10212), using insertion sites identified by intronserter (Jaeger et al., 2019). The coding sequence of Afr-10212 used for intron insertion is provided in the Extended Data. The IR98 gene contained two introns and thus no further modifications were necessary. The SL1 sequence (5'-TTTCAG-3') was added upstream of the start codons, creating *Afr* -opt-RFP and *Afr* -opt-Hyg; these sequences were synthesized as gBlock fragments (IDT). Plasmid pAS66 was generated by replacing the *Ppa* -RFP gene in pAS19 (Adams et al., 2019) with *Afr* -opt-RFP. Plasmid pAS71 was constructed by inserting *Afr* -opt-Hyg between the *Arh- rps-0* promoter (g9100.t1) and the *Arh- tbb-2* 3'UTR region from pAS19 (Tandonnet et al., 2019) in a pGEMT easy backbone (Promega).

The pMJ5 plasmid was constructed by inserting the hygromycin B resistance gene cassette from pSNP44 (Namai and Sugimoto, 2018) and the transgene ( *Ttu- myo-2 p::gfp::Ttu- myo-2* _3'UTR) into the pPD95_75 vector (a gift from Andrew Fire, Addgene # 1494). The complete nucleotide sequences of plasmids pAS66, pAS71, and pMJ5 are included in the Extended Data.

**Microscopy**

Fluorescent images were captured using a DIC microscope (Olympus BX53) equipped with a Hamamatsu ORCA-spark camera and an Olympus U-HGLGPS fluorescence light source. Images were processed using Olympus cellSens software.

**Protocol for microparticle bombardment and hygromycin B screening**

The protocol for microparticle bombardment and hygromycin B screening for this study was based on the protocols for *C. elegans* (Schweinsberg and Grant, 2013) and *P. pacificus* (Namai and Sugimoto, 2018) with some modifications for *A. freiburgense***** and *T. tufae* .

**I. Growing large quantities of nematodes**

Mass worm cultures were grown in 20 large (90 mm) Petri dishes containing enriched agar medium (0.3% NaCl, 0.5% Polypeptone, 0.1% dried yeast extract, 2.5% agar, 5 µg/mL cholesterol, 1 mM CaCl _2_ , 1 mM MgSO _4_ , and 2.5 mM K phosphate buffer) plates and seeded with 1 mL of *E. coli* OP50 . For *A. freiburgense* cultivation, 20 unit/mL Nystatin was added. Cultures were maintained at 25 °C. About 100,000 adult worms are necessary for bombarding one construct. Synchronization of cultures was necessary.

**II. Synchronizing *T. tufae* adult worms**

1. Resuspend a culture with mixed stages in 1% SDS dissolved in M9 buffer (22.0 mM KH _2_ PO _4_ , 63.4 mM Na _2_ HPO _4_ , 85.6 mM NaCl) for 10 min. This treatment will dissolve all worms except the dauer larvae.

2. ​​Wash the isolated dauer larvae three times with M9 buffer.

3. Plate ~100,000 dauer larvae onto 90 mm NGM plates seeded with *E. coli* OP50 . Incubate at 20 °C for 44-48 hours to obtain young adults containing non-hatched offspring. To prevent starvation, culture the worms on multiple plates as needed.

4. Collect the synchronized adult worms in M9 buffer into a 50 mL conical tube.

5. Centrifuge at 2,000 rpm for 1 min. to pellet the worms and remove the supernatant.

**III. Washing gold microcarriers**

**​​** Weigh 60 mg of 1.6 μm Gold Microcarriers (Bio-Rad, 165-2264) into a 1.5 mL siliconized tube and vortex.

1. Add 1 mL of 70% ethanol and vortex for 5 min.

2. Settle for 15 min. at room temperature.

3. Centrifuge the tube for 20 sec. at 6,000 rpm and remove the supernatant.

4. Add 1mL of distilled water and vortex for 1 min.

5. Settle for 1 min. at room temperature.

6. Centrifuge the tube for 20 sec. at 6,000 rpm and remove the supernatant.

7. Repeat steps 4-6 three times to wash the gold microcarriers.

8. Suspend the gold microcarriers in 500 μL of 50% glycerol and store at 4 °C. (The gold microcarriers in 50% glycerol can be stored for 2 months.)

**IV. Coating DNA onto gold microcarriers**

1. Vortex the washed gold microcarriers for 10 min.

2. Immediately, transfer 400 μL of the gold microcarriers in 50% glycerol to a new siliconized 1.5 mL tube and vortex for 1 min.

3. For *T. tufae* , add 50 μL of 300 ng/μL DNA, 150 μL of 2.5 M CaCl _2_ , and 60 μL of 0.1 M spermidine in order. For *A. freiburgense* , use either 40 µg of a single plasmid, or 40 µg per plasmid when working with two plasmids.

4. Vortex for 5 min.

5. Centrifuge the tube for 20 sec. at 6,000 rpm and remove the supernatant.

6. Add 300 μL of 70% ethanol and vortex the tube for 5 min.

7. Centrifuge the tube for 20 sec. at 6,000 rpm and remove the supernatant.

8. Add 170 μL of 100% ethanol and vortex the tube for 5 min.

9. Use the gold microcarriers immediately for microparticle bombardment.

**V. Microparticle bombardment**

Microparticle bombardment was performed using Biolistic PDS-1000/He Particle Delivery System (Bio-Rad, 165-2257) with Hepta Adaptor (Bio-Rad, 165-2225), 1550 psi Rupture Disks (Bio-Rad, 165-2331), and Hepta Stopping Screens (BioRad, 165-2226).

1. Wash seven macrocarriers (Bio-Rad, 165-2335) with 70% ethanol and let them air dry.

2. Place a macrocarrier into a Hepta Adaptor.

3. Load 20 μL of gold microcarriers onto each macrocarrier and air-dry.

4. Soak Rupture Disks (1550 psi) in 70% ethanol and let them air dry.

5. Place a washed Rupture Disk in the Hepta Adaptor.

6. Spread 1 mL of worm suspension (~20,000 worms) uniformly onto a well-dried and cooled 90 mm NGM plate.

7. Perform microparticle bombardment according to the manufacturer's instructions, and set the bombardment chamber's negative pressure to 28 inches of Hg.

**VI. Recovery after microparticle bombardment**

1. Add M9 buffer onto each bombarded plate and resuspend the worms.

2. Distribute the worm suspension onto 5 NGM 90 mm plates seeded *E. coli* OP50 .

Incubate the plates for 24h at 25 °C for *A. freiburgense* and four days at 20 °C for *T. tufae.*

**VII. Hygromycin B selection**

1. Add the 10 mg/mL hygromycin B (Invitrogen) onto the plates onto the plates to a final concentration of 300 μg/mL and let them dry.

2. Incubate the plates with *A. freiburgense* at 25 °C and *T. tufae* at 20 °C for 2 weeks. Add LB (85.6 mM NaCl, 0.01% Tryptone (Gibco), 0.005% Yeast extract (Gibco), pH to 7.0 using 1 M NaOH) with *E. coli* OP50 to avoid the worms to starve.

3. Two weeks after the bombardment, screen for survivors.

## Reagents

**Table:** 

***A. freiburgense***		
**strain**	**genotype**	**Available from**
APS30	*brzls1 [Arh-eft-3p::Afr-RFP::Afr- tbb-2 _3'UTR + Arh- rps-0 ::HygR:: Arh- tbb-2 _3'UTR] *	This work
APS31	*brzls2 [Ttu- myo-2 p::GFP::Ttu- myo-2 3'UTR::Ttu- rps-0 p::HygR::Ttu- rps-0 _3'UTR] *	This work
APS32	*brzls3 [Ttu- myo-2 p::GFP::Ttu- myo-2 3'UTR::Ttu- rps-0 p::HygR::Ttu- rps-0 _3'UTR] *	This work

**Table:** 

***T. tufae***		
**strain**	**genotype**	**Available from**
SHR201	*amu10* [ *Ttu- myo-2 p::gfp::Ttu- myo-2 _3'UTR;Ttu- rps-0 p::HygR::Ttu- rps-0 _3'UTR* ]	This work

**Table:** 

**Plasmids for microparticle bombardment**		
**Plasmid**	**Genotype**	**Description**
pAS66	* Arh-eft-3p::TurboRFP::Arh- tbb-2 _3'UTR *	Promoter and 3'UTR derived from *A. rhodense .* TurboRFP is codon-optimized for *A. freiburgense . *
pAS71	* Arh- rps-0 p::HygR::Arh- tbb-2 _3'UTR *	Promoter and 3'UTR derived from *A. rhodense .* HygR is codon-optimized for *A. freiburgense . *
pMJ5	* Ttu- myo-2 p::gfp::Ttu- myo-2 _3'UTR;Ttu- rps-0 p::HygR::Ttu- rps-0 _3'UTR *	Promoter and 3'UTR derived from *T. tufae.* GFP and HygR are not *T. tufae* -optimized.

## Data Availability

Description: Afr-actin_1 (Afr-10212) sequence. Resource Type: Dataset. DOI: https://doi.org/10.22002/dxera-hze30 Description: pAS66 sequence. Resource Type: Dataset. DOI: https://doi.org/10.22002/rjk5n-sm989 Description: pAS71 sequence. Resource Type: Dataset. DOI: https://doi.org/10.22002/98ycf-p9j66 Description: pMJ5 sequence. Resource Type: Dataset. DOI: https://doi.org/10.22002/4jx5g-y7v88
